# Trade-offs between vocal accommodation and individual recognisability in common marmoset vocalizations

**DOI:** 10.1038/s41598-021-95101-8

**Published:** 2021-08-03

**Authors:** Y. Zürcher, E. P. Willems, J. M. Burkart

**Affiliations:** Department of Anthropology, University of Zürich, Winterthurerstrasse 190, Zurich, Switzerland

**Keywords:** Biological anthropology, Animal behaviour

## Abstract

Recent studies find increasing evidence for vocal accommodation in nonhuman primates, indicating that this form of vocal learning is more prevalent than previously thought. Convergent vocal accommodation (i.e. becoming more similar to partners) indicates social closeness. At the same time, however, becoming too similar may compromise individual recognisability. This is especially problematic if individual recognisability is an important part of the call function, like in long-distance contact calls. In contrast, in calls with a different function, the trade-off between signalling social closeness and individual recognisability might be less severe. We therefore hypothesized that the extent and consequences of accommodation depend on the function of a given call, and expected (1) more accommodation in calls for which individual identity is less crucial and (2) that individual identity is less compromised in calls that serve mainly to transmit identity compared to calls where individual recognisability is less important. We quantified vocal accommodation in three call types over the process of pair formation in common marmoset monkeys (*Callithrix jacchus*,* n* = 20). These three call types have different functions and vary with the degree to which they refer to individual identity of the caller. In accordance with our predictions, we found that animals converged most in close contact calls (trill calls), but less in calls where individual identity is more essential (phee- and food calls). In two out of three call types, the amount of accommodation was predicted by the initial vocal distance. Moreover, accommodation led to a drop in statistical individual recognisability in trill calls, but not in phee calls and food calls. Overall, our study shows that patterns of vocal accommodation vary between call types with different functions, suggestive of trade-offs between signalling social closeness and individual recognisability in marmoset vocalizations.

## Introduction

Nonhuman primates hardly learn new call types, neither as infants nor as adults^1^, and were thus often considered to lack vocal production learning altogether. However, according to the definition by Janik and Slater^2^, vocal production learning also occurs when vocal signals are modified due to the experience with those of another individual, and thus also includes the modification of calls already existing in the repertoire of an individual. This form of vocal learning, called vocal accommodation, is quite abundant in nonhuman primates, can be found in different species and contexts, and often functions to signal social closeness in nonhuman and human primates alike^3^. Social vocal accommodation can take the form of convergence (becoming more similar) and divergence (becoming more different) and has been found in a variety of situations^3^. Japanese macaques show increased vocal accommodation to higher ranking individuals^4^, both Diana monkeys^5^ and chimpanzees converge towards communication partners in a short time range^6^, and Campbell’s monkeys share more similar call variations with individuals with whom they have stronger social bonds^7^. Ample evidence for vocal learning in the form of vocal accommodation has also been reported in the callitrichidae, a primate family known for its vocal flexibility^8–11^. In a colony of pygmy marmosets (*Cebuella pygmaea*), the introduction of unfamiliar individuals led to a shift in the call range of all the individuals^12^, and Wied’s black-tufted-ear marmosets (*Callithrix kuhlii*) were found to modify their call structure after unfamiliar individuals were introduced into the colony room^13^. In a recent study on common marmosets, individuals became more similar to a new colony after having been translocated from a colony with a different vocal variant^14^, and the change of vocalization was most likely due to social vocal learning rather than changes in the environment^15^.

Vocal learning in the form of vocal accommodation is thus common in nonhuman primates (and quite likely also in other animals^3^) and often seems to serve a social function^16^. Vocal convergence has been suggested to play a role in group cohesion, could lead to a group signature, facilitate recognition of group members, and is often linked to pair bond quality^3,16^. However, a hitherto neglected aspect of vocal convergence is that it may also have a less desired consequence: converging towards a communication partner may reduce the individual recognisability of a vocalization. This will lead to a trade-off between the need to accommodate as a social signal, and the need to encode identity in calls. Such a trade-off predicts a “sweet spot”, basically the vocal distance between communication partners that animals should aim for where both the specific needs for accommodation and individuality are in equilibrium. Depending on call type and associated function, this equilibrium can be at a relatively larger or smaller vocal distance between communication partners.

**Figure 1:**
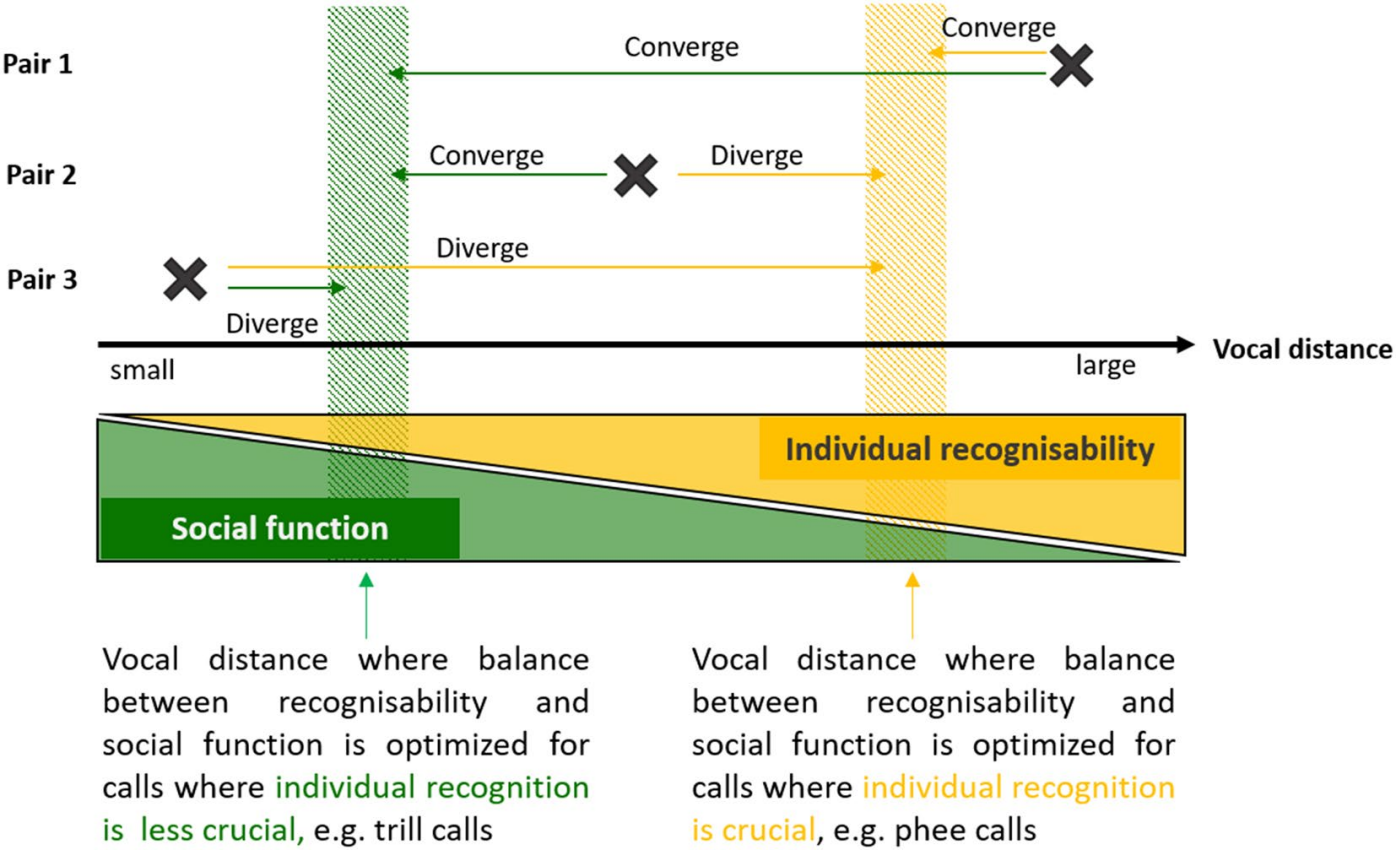
Schematic representation of the predicted trade-off between accommodation and individual recognisability. Depending on the initial vocal distance and call type, patterns of accommodation are expected to vary in different pairs, as exemplified by pair 1–3. Orange arrows indicate the amount and direction of accommodation in a pair for a call where individual recognition is crucial, green arrows for calls where individual recognition is less important. If animals are very similar (pair 3) or very different (pair 1) prior to pair formation, convergence or divergence might be found in any call type, but to a different degree. If a pair shows intermediate vocal distance (pair 2) the pattern of accommodation might vary depending on the call function. Coloured triangles at the bottom represent the amount of social function (green) or individual recognisability (orange) that can be represented in a call at any given vocal distance (black arrow) between two individuals. If individual recognition in a call is important, the optimal distance should be where individual recognition is still strong (orange arrow and orange shaded area). If individual recognition is less important, the optimal vocal distance is expected where the social function is making up a larger part (green arrow and green shaded area).

We studied accommodation in newly formed pairs of common marmosets and predicted that the amount of convergence should differ between call types with different functions. In call types for which individuality is less important, such as close distance calls, more convergence might take place, while in call types for which transmitting individuality is paramount, as for instance in long-distance contact calls that are given out of visual contact, convergence should be constrained by the necessity to maintain individual recognisability. Thus, for dyads that want to signal social closeness, as in newly established breeding pairs, there should be an optimal vocal distance for each call type, at which the animals can signal social closeness without compromising the necessary amount of individual recognisability (Fig. 1). As a consequence, the amount and direction of accommodation during pair formation should depend on the initial vocal distance between partners. Pairs whose initial vocal distance is larger than the optimal distance should converge over the time of pair formation, whereas pairs who happen to have vocalizations that are more similar than the optimum should diverge (see Fig. 1). Consistent with this idea, Snowdon and Elowson found vocal convergence in pygmy marmosets in only 3 out of 4 newly formed breeding pairs; the individuals of the fourth pair who did not converge were very similar to each other already before pair formation^17^.

A second set of predictions follows from a hypothesised trade-off between accommodation and individuality and concerns the impact of accommodation on individual recognisability. In particular, accommodation should differently impact individuality depending on call function. In call types such as long distance contact calls for which transmitting identity is essential, animals should converge little and in particular not at the expense of call individuality. In short distance contact calls however, where individual identity is less important because callers are typically within visual contact, there should be more flexibility to engage in accommodation, which can be achieved at the expense of individual recognisability. A similar argument was previously made in a study on individual baboon distress- and contact calls, in which the latter were found to contain a stronger individual signature than the former^18^. Moreover, the authors of this study suggested that a call type with fewer functional constraints might be structurally more free to vary to convey individual identity, which might be true for vocal accommodation as well.

To investigate potential trade-offs between accommodation and individuality, we measured the vocal output of a total of 20 common marmosets in ten newly formed breeding pairs over the process of pair formation for seven to twelve weeks. Several species of marmosets are known to show a high degree of vocal flexibility and a certain degree of vocal learning including babbling in infants^19–21^, the importance of social input for vocal ontogeny, including feedback by parents^22–24^ and acoustic differences between colonies or populations^14,25–27^. We analysed three different call types with different social functions (phee calls, trill calls and food calls) that are regularly produced in a within-group (i.e. breeding pair) context. We first investigated the extent of accommodation within each dyad for each call type, by quantifying both vocal convergence, and divergence. Next, we tested whether the amount of accommodation was correlated with vocal distance prior to pair formation for each call type. Finally, we statistically tested how accommodation impacted the potential recognisability of individuals.

Phee calls are predominantly used as long distance isolation calls when individuals are separated from their mates or social group^28,29^. Phee calls are known to encode individual identity as well as group identity and sex^30–32^. They typically elicit answering phee calls from other group members or mates, and animals often engage in turn taking, i.e. calling back and forth over several turns^33^. Common marmosets also use phee calls as vocal territory advertisement^34^, although the social situation seems not necessarily to be encoded in the call^32^. Overall, these functions suggest that individual recognisability is essential in phee calls.

Trill calls are close distance social calls that are often exchanged between social partners in a very relaxed social situation. Wild common marmosets often produce trill calls in situations such as foraging or resting^28^. A study by Liao et al. could show that captive marmosets produce trill calls more often when being closer to their social partner and with a lower heart rate, so at a more relaxed state, compared to when producing phee calls^29^. Since trill calls are given from a close distance, typically even within visual range, we assume that individual recognisability is less essential than in phee calls.

The third call type we looked at were food calls (sometimes also referred to as chirp calls)^28^. Food calls are usually produced upon the detection of high value food and often indicate the willingness of the caller to share the food with other group members^35^. Food calls are usually produced in bouts, and are given from variable distances. They seem to be more variable than trill calls and phee calls, and might have some elements that are referential with regard to food type^36^. Food calls typically inform infants and juveniles about the presence of food items, which are subsequently offered to them by the caller^37^, but are also used by pair members (often the male) towards their mate^38^. Food calls and food sharing between adults might facilitate the development of a pair bond^39^, which is why it might be of specific interest in newly bonding animals. As marmosets can be rather dispersed during feeding, recipients may not be in the immediate vicinity of the group member producing food calls, and therefore, a clear signal of individual identity could help receivers to move towards the caller to receive the food. Table 1 provides an overview of all the predictions for the specific call types.

**Table 1:** Overview of the predictions: the table provides a short description of the three call types and how important transmitting identity (ID) is for each of them, and the predictions with regard to the patterns and consequences of accommodation.

	Phee calls	Trill calls	Food calls
Call description	Long distance contact calls, produced when separated, out of sight	Close distance contact calls, usually produced within sight of others	Communicates detection and intent to share food; with or without close contact
Importance of ID	Crucial, as animals have to convey their ID	Less important; redundant as ID is directly visible	Important for receiver to know which individual is willing to share food
**Predictions: patterns of accommodation**			
Extent of accommodation	Little accommodation expected	Highest amount of accommodation expected	Little accommodation expected
Accommodation correlates with initial vocal distance	Expected	Expected	Expected
**Predictions: consequences of accommodation**			
Reduction in statistical individual distinctiveness	Not expected	Possible	Not expected

## Methods

### Subjects

We recorded the vocal behaviour of 20 captive common marmosets over the process of pair formation of newly formed breeding pairs. All animals lived with at least one family member or a former partner until shortly before we introduced to their new breeding partner. After the animals were introduced to their new partner, they were no longer in acoustic contact with their former family or mate, but could hear other marmoset groups that were housed in the same room. Animals ranged from between 2 to 9 years, and all individuals were unfamiliar with their new partner before the start of the study.

The enclosure of each pair measured 2.4 m in height × 1.5 m in depth × 0.8 m in width and was structured with branches, ropes, tubes and other enrichment material. All animals were fed twice a day (vitamin enriched mush in the morning and a mix of fruits and vegetables around midday) and in addition received different kind of animal or insect protein and/or gum once to twice a day. Water was always available ad libitum. The animals had regular access to spacious outdoor enclosures as well as to an additional testing room.

### Recording procedure

The animals were recorded both before and during pair formation in a variety of situations to elicit a broad range of calls covering a large part of the naturally occurring call spectrum of the marmoset (presentation of food to elicit food calls, recordings with partner to elicit trill calls, recordings when separated from the partner to elicit phee calls). Before pair formation, individuals were recorded on several days over two to three weeks in their home enclosure either with a family member present or after being separated from their family group, as closely in time to pair formation as possible. After pair formation, we recorded the animals on one to three days a week up to 13 weeks after pair formation. We recorded them both in their home enclosure and in an additional, familiar experimental room which was connected to the home enclosure by a system of tubes through which the animals could walk. When recorded in their home enclosure, both animals of the pair were present. When recorded in the additional testing room, animals were either both present or they were separated from each other (either with the other animal still in the room with acoustic contact, or with the other animal back in the home enclosure) for up to five minutes. Both in the home enclosure and the test room, animals were recorded with or without highly preferred food (a mixture of mealworms, cashew seeds and nut-cookies). Recording sessions lasted between 20 and 30 min. During the recording, the experimenter was present in the room and pointed the hand held microphone in the direction of the focal animal, which changed every five minutes. The identity of the caller was directly annotated to the recording by the experimenter in real time using the labelling function provided by the AviSoft Recorder software^40^.

Even though we tried to elicit calls from the animals, data recording remained largely opportunistic. Therefore, we do not have all call types of all the individuals over the whole time period. Pairs with less than a minimum of five calls per call type and per point in time where therefore excluded from further analysis, which led to a final sample of 8–9 pairs, depending on the call type.

The study and all the proceedings were reviewed and approved by the Kantonales Veterinärsamt Zürich, licence number ZH223/16 and followed both the ARRIVE guidelines as well as all other important guidelines and regulations.

### Recording processing

The recordings were visually inspected in AviSoft Pro^40^ and each call saved as a separate file. We inspected and measured each call with the software Praat^41^ and extracted 15 (phee, food call) or 17 (trill) parameters per call after a script by E.F. Briefe & A. G. McElligot^42^. We measured the fundamental frequency and extracted the frequency both at the beginning and the end of the call, further the mean, minimal and maximal F0, the percentage of the call duration for which F0 was at the max, the absolute slope of F0, the mean variation of F0 per second, the frequency values at the first, second and third quartiles of energy, the highest frequency of the whole spectrum, percentage of time this highest frequency is reached and jitter, as well as frequency modulation rate and frequency modulation extent for trill calls (see Ref.^14^ for a detailed description of the parameters). Calls were excluded from the final sample if there was background noise, if they overlapped with any other call or we could not measure the whole call correctly in Praat.

### Statistics

#### Patterns of accommodation

To quantify convergence and divergence, we calculated the vocal distance between partners before the start of pair formation (bpf) and after pair formation (apf) for each call type (see Table 2 for the specific time after pair formation the apf—calls were recorded per pair and call type). We first performed principal component analyses for each call type and each pair based on the z-transformed values of the measured call parameters and extracted all components with an Eigenvalue greater than the 95% quantile value obtained from 10,000 datasets that were randomly generated and equal in sample size and dimensionality to our empirical data (Parallel analysis). This lead to 3–5 extracted factors depending on pair and call type. For all further analyses, we used the PC-Factors extracted by this method.

**Table 2:** Amount of accommodation (convergence and divergence) for each pair and call type. Week refers to the week after pair formation when the recordings for the “after”-comparison were made (for phee-/trill-/ and food calls respectively). *α-level* gives the level at which the vocal distance was significantly different before and after pair formation (ns indicates that the change in distance was not significant). *r* indicates the effect size, while the + or − indicates the direction of the effect. *Positive r values* indicate convergence, i.e. that the pair became more similar, *negative r values* indicate divergence.

Pair	Week last recording (phee/trill/food)	Phee call		Trill call		Food call	
		α-level	r	α-level	r	α-level	r
WiscoNaut	10–11/9/11	0.05	+ 0.131	0.001	+ 0.229	0.01	+ 0.314
WashGatto	10–12/9/10	ns	+ 0.136	0.001	+ 0.141	0.01	− 0.294
NikPuk	10/9–10/–	0.05	+ 0.148	0.001	+ 0.254	–	–
MibbCon	9–10/7–9/9	0.05	− 0.322	ns	+ 0.004	0.05	+ 0.071
LilCrak	9–13/9/9	0.05	+ 0.058	ns	− 0.039	0.01	− 0.165
NalaTam	6–7/6/6	ns	+ 0.019	ns	− 0.010	0.01	+ 0.353
LeaKyr	6–9/7/9	0.05	+ 0.148	0.001	+ 0.318	0.01	− 0.204
MiaNari	10/7/10	–	–	0.05	+ 0.101	0.01	− 0.110
TogaMio	10/7/9	ns	+ 0.064	ns	+ 0.035	0.01	− 0.194
JajaMembo	–/–/9	–	–	–	–	0.01	− 0.184

We calculated the Euclidian distance between each call of the male and each call of the female within a pair based on the extracted PC-factors. It is important to note here that—as each call served as a reference for multiple distance measurements (each call was compared to each call of the partner)—these distance measurements between partners are not independent, and this non-independence has to be taken into account in the analysis. To estimate whether the vocal distance increased or decreased over time in the different pairs, we compared the distance matrix bpf with the distance matrix apf with a bootstrapped Welch t-test (taking into account the dependencies in the data) and calculated non-parametric 95–99.9% confidence intervals around the effect size to assess whether there was a significant change in the vocal distance. An increase in distance would indicate vocal divergence, a decrease in distance vocal convergence. We used the average of the Euclidian distances as a proxy for average vocal distance between partners for either point in time. The amount of accommodation was calculated as the change in vocal distance bpf to apf by subtracting the average vocal distance apf from the average vocal distance bpf. We calculated Pearson’s correlation coefficients to test if the initial distance between pair mates and the amount of accommodation was correlated, separately for each call type.

#### Impact on statistical individual recognisability

We investigated whether animals could statistically be distinguished by their calls, and whether this changed with accommodation. We first again performed a PCA as described above, this time including the calls of all the individuals in one analysis. We then performed a Discriminant Function Analysis (DFA) both before and after pair formation to quantify to what extent calls could statistically be correctly assigned to the individual producing them, using the total of the correctly assigned calls as a measure of individual distinctness within calls. To test whether the amount of correctly assigned calls changed from before to after pair formation we performed a binomial GLMM, including “condition × call type” as fixed effects and “individual nested in pair” as well as “call type” as random effects. Lastly, we compared the mean of correctly assigned calls between bpf and apf split by call type using post-hoc comparisons (function “emmeans”, package “emmeans”). All analysis were performed in R 3.5.3.

## Results

### Patterns of accommodation across call types

**Figure 2:**
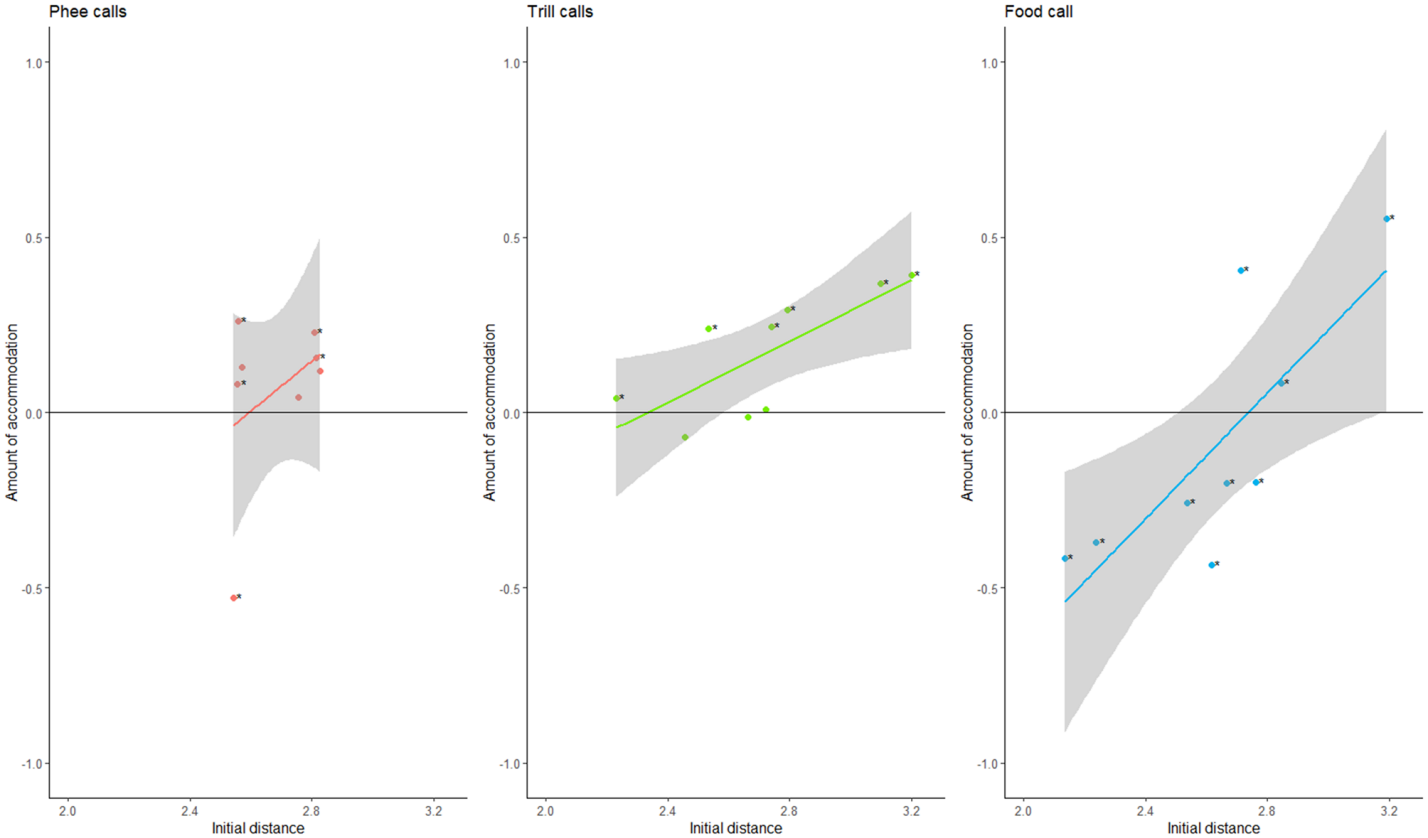
Relationship between the amount of accommodation and the initial vocal distance of each pair for phee-, trill-, and food calls. Significant changes in vocal distances are indicated with an *. *Positive accommodation* indicates convergence, i.e. that the pair became more similar, *negative accommodation* indicates divergence. For both trill calls and food calls, the amount of accommodation was predicted by the initial distance.

To disentangle how the calls changed over time, we quantified the amount of accommodation (both convergence and divergence) for each pair and each call type. We found that for phee calls, 5 out of 8 pairs showed a significant amount of accommodation, of which 1 pair diverged and 4 pairs converged. In trill calls, 5 out of 9 pairs showed a significant amount of accommodation, all of which converged. In food calls, all 9 pairs showed a significant amount of accommodation, and 3 pairs converged, while 6 pairs diverged (see Table 2, Fig. 2). Convergence was thus most prevalent in trill calls (55.56% of all pairs), followed by phee calls (50%) and food calls (33.33%).

Next, we tested whether the amount of accommodation was correlated with the initial vocal distance of the individuals before pair formation. While in phee calls we did not find a significant effect of the initial call distance on accommodation, even though effect size was medium to large (N = 8, Pearson’s correlation coefficient = 0.381, p = 0.352), both trill calls (N = 9, Pearson’s correlation coefficient = 0.744, p = 0.022) and food calls (N = 9, Pearson’s correlation coefficient = 0.782, p = 0.013) showed a positive correlation between the initial vocal distance and the amount of vocal accommodation (see Fig. 2).

### Impact of accommodation on statistical individual recognisability

**Figure 3:**
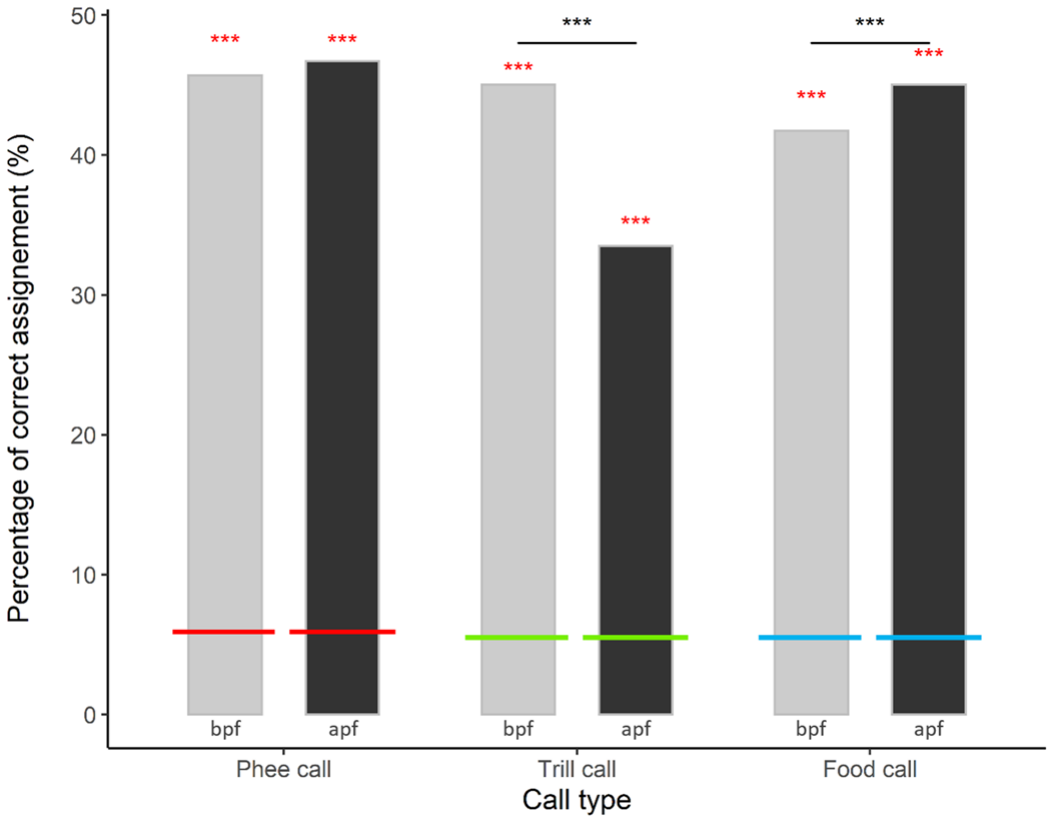
Statistical individual recognisability before and after pair formation. Percentage of correct assignments was obtained from a discriminant function analysis. Light grey bars indicate values of correct assignment before pair formation, dark grey bars after. Calls can be attributed to the correct individual by discriminant function analysis significantly better than expected by chance (red, green or blue line respectively, indicated by red asterisk) in all conditions. The amount of correct assignment though significantly decreased in trill calls after pair formation and increased in food calls (GLMM, indicated by black asterisk). We did not observe a significant change in the level of correct assignment in phee calls.

To quantify the impact of the observed patterns of accommodation on statistical individual recognisability, we compared the amount of calls correctly classified to individuals before and after pair formation. The expected amount of correct classification by chance was around 6% for each call type, and calls were always correctly classified to higher amounts than expected, i.e. statistical individual recognisability was high in each call type (Fig. 3). When performing a discriminant function analysis, statistical individual recognisability remained at comparable levels before (45.7%) and after (46.7%) pair formation for phee calls. In trill calls, statistical individual recognisability significantly dropped from 45% (bpf) to 33.5% apf, and in food calls, it was slightly increased apf (41.7% bpf vs. 45% apf) (Fig. 3). The GLMM shows a significant difference between the call types and the situation (bpf vs apf) (Table 3). Post hoc tests revealed that the changes in statistical individual recognisability were significant in both trill calls and food calls (Table 4).

**Table 3:** The analysis of deviance table (Type II) for the binomial GLMM shows that the effect of pair formation on the correct assignment of calls to individuals, was call type specific. Significant (highest-order) effects are indicated with p-values in bold.

Response: correctly classified calls			
	Chi^2^	df	p
Condition [(bpf) vs. (apf)]	7.36	1	0.007
Call type	26.03	2	2.224e−06
Condition × call type	63.58	2	**1.559e−14**

pseudo-R^2^ = 0.25, N_observations_ = 106 on N_Individuals_ = 20,in N_Pairs_ = 10; N_Call types_ = 3.

**Table 4:** Post hoc tests reveal that while trill calls are significantly less likely to be correctly assigned to the correct individual after pair formation than before, the probability of correctly assigning food calls to the correct individual is higher after pair formation than before. There was no difference in correct assignment to be found in phee calls. p-values are Tukey HDS corrected to take multiple testing into account.

Contrast	Call type	Estimate	SE	z-ratio	p-value
Correctly assigned calls bpf vs apf	Phee call	− 0.0223	0.0870	− 0.257	0.7973
	Trill call	− 0.4675	0.1180	− 3.960	0.0001
	Food call	0.629	0.0853	7.380	< 0.0001

## Discussion

Increasing evidence for vocal accommodation in nonhuman primates has received a lot of attention in recent research because it suggests more vocal learning than previously assumed. When vocally accommodating, animals modify their vocalizations due to a social template, satisfying the definition of vocal learning by Janik and Slater^2^. Vocal accommodation often seems to serve a social function, reflecting social distance or the strength of a social bond. Nevertheless, an excess in vocal convergence can have disadvantages, when increasing vocal similarity leads to a loss in individual recognisability^43^. In this study, we explored potential trade-offs between the social benefits of convergence vs the necessity to maintain individuality in call structure in common marmosets. To do so, we tested newly formed pairs and compared their vocalizations before and after pair formation. This situation has elicited vocal accommodation in pygmy marmosets previously^17^, but so far it was unclear whether and how marmosets would deal with the different requirements of converging to a partner while keeping their identity encoded in the calls. In this study, we therefore investigated how common marmosets accommodate to their partners in three different call types that critically differ in their function: Phee calls, which are long distance contact calls mainly produced when animals are separated from social partners; trill calls, which are close distance calls usually produced in close proximity, and food calls, which are emitted when animals find preferred food, often indicating willingness to share. In a second step, we examined to what extent their pattern of accommodation impacted how well calls could be attributed to specific animals statistically (individuality of calls), and whether this was related to the different call functions.

### Patterns of accommodation across call types

In our first set of predictions, we expected that the amount of convergence should differ between call types with different functions if there is a trade-off between the social function of accommodation and individual identity. We found vocal accommodation in all three call types, but to a different degree. As predicted, most convergence was observed in the close-distance trill calls, and less in long distance phee- and food calls. These results are in line with studies in other marmoset species, which found that animals show vocal accommodation in their trill calls in different situations^12,13,15,17^. In trill calls we only found convergence, whereas in phee calls and food calls we found both convergence and divergence. Further, we found that in trill calls and food calls, the amount of accommodation was correlated with the initial vocal distance between pairs, but not in phee calls. From our data, we cannot conclude that such a correlation is really absent in the phee calls, or if failing to reach significance is, as suggested by the rather large effect size, an artefact of the rather small sample size. Over all, these results fit the hypothesis that a trade-off between social accommodation and preserving individual identity leads to different patterns of accommodation depending on the call function (i.e. how important it is that individual identity is encoded in a specific call type) as well as the idea of an optimal vocal distance between partners where the benefits of accommodation are reached but the negative impacts minimized. To further test this idea, in a next step we investigated whether these differences in accommodation pattern indeed affected the individual recognisability of call depending on the call types.

### Consequence of accommodation for individual recognisability

Next, we investigated how well calls can be individually distinguished with statistical methods. In trill calls, where individuality is less important and which showed the highest level of convergence, we found a significant *decrease* in the individuality of the calls (calls could be assigned to the correct individual less reliably). In phee calls, where individuality is crucial, the statistical individual recognisability *did not change* even though convergence occurred in some pairs. In food calls, where individuality is also important and where divergence was most prevalent, calls could be *better* assigned to the correct individual after pair formation. It therefore seems that convergence did indeed reduce individual distinctness only in the call type (trills) where it is less important because the animals can see each other directly when emitting such calls. In our study, we unfortunately could not look into how changes in statistical recognisability impacted caller recognition by the animals themselves. Playback experiments would therefore be an important next step to investigate whether our findings also impact the ability of the receivers to distinguish between callers. Additionally, presenting playbacks that simulate potential partners with more or less similar calls, could answer the question if and how common marmosets use potential information encoded in different call types. Our results though show that vocal accommodation seems to be regulated differently for individual call types and is probably a more complex process than hitherto expected. How convergence is differently regulated in phee- and food calls compared to trill calls remains to be established.

Based on our predictions (Table 1), we would have expected similar results in both phee- and food calls. Whereas convergence occurred in all three call types, divergence occurred in food calls in particular. So what differentiates this call type from the others, especially from phee calls? In contrast to food calls, phee calls are also produced in inter-group encounters, and are known to be group-specific to a certain degree. This might limit their potential to diverge between partners in addition to the constraints already discussed. Further, the food calls of the future pairs were potentially rather similar already before pair formation, which arguably led to this high level of divergence. It thus appears that individual recognisability is indeed important for food calls, and future studies using playbacks will help disentangle why this is the case.

What we did not consider in this study is the fact that food calls are normally produced in call bouts that contain several individual food call elements. In our analysis, we only analysed the single elements but not the information that is potentially encoded in the call bout. An intriguing possibility is that marmosets also accommodate to their partner with regard to bout structure (e.g. duration, number of elements), similar to the occurrence of accommodation in humans at multiple levels, from acoustic structure to word choice and syntax^3^. Moreover, some elements of marmoset food calls appear to be functionally referential^36^. Taken together, the food calls thus appear more heterogeneous than the other two call types analysed here, and additional studies will be necessary to fully understand how they change together with changes in social context.

Our main research focus of this study was to establish how the different needs for accommodation and individuality can be accounted for. It therefore provides an important background for other studies on vocal accommodation to come. Whether or how vocal similarity or dissimilarity is used as a social signal in common marmosets is still an open question, both in breeding pairs as we studied them, as well as in the larger family groups marmosets usually live in. Based on studies in other animals, it is well possible that accommodation, or another means of vocal flexibility, is used by common marmosets to signal the strength or even maintain their pair bond^3^. We can only speculate though whether vocal similarity indeed strengthens social bonds between all individuals in a group, as it would be equally important between breeding and non-breeding group members^44^. We would consider it likely though, especially if groups contain non-related helpers, where kin selection is not sufficient to ensure cooperation. Our results suggest that trill calls are particularly likely candidate vocalisations for such a function, as they are more prone to accommodation and appear less constrained by the need to maintain individual recognisability. Moreover, they are often produced by animals which are in close contact and have a strong social bond^29^.

Vocal learning was for a long time considered rare in nonhuman primates^1^. In this study, we could confirm that common marmosets engage in vocal accommodation—a form of vocal learning—quite regularly—but also, that they most likely face trade-offs between similarity and individuality. Together, this corroborates that common marmosets have a high level of vocal flexibility, and that they use vocal accommodation as a very flexible tool which appears regulated differently depending on call types and call type functions.
